# Optimization of heat shock, acid shock and salt stress process and its mechanism of protection before spray drying of Baijiu yeast - *Modified sporidiobolus Johnsonii A*

**DOI:** 10.1186/s40643-025-00939-9

**Published:** 2025-11-05

**Authors:** Fengkui Xiong, Zhongbin Liu, Jingyu Li, Guangzhong Hu, Duqing Qu, Bo Huang

**Affiliations:** College of mechanical engineering, Library, Sichuan University of science & engineering, Yibin, 644000 China

**Keywords:** Spray drying, *Modified sporidiobolus johnsonii A*, Acid shock, Heat shock, Salt stress, Protection mechanism

## Abstract

The low survival rate of high-quality baijiu yeast, *Modified Sporidiobolus Johnsonii A* (MSJA), during spray drying and the unclear mechanisms underlying its stress treatment processes (heat shock, acid shock, and salt stress) have significantly hindered its low cost and high effective application, thereby impacting the high-quality development of the liquor industry. To address this problem. A response surface experiment was first conducted to optimize the stress treatment process prior to spray drying. Subsequently, the mechanisms and efficacy of the stress treatments were analyzed using Field Emission Scanning Electron Microscopy (FESEM). The results demonstrated that the survival rate of spray-dried MSJA could be improved from 38.5 to 54.8% under the optimal conditions, with heat shock temperature (X) = 35.2 °C, acid stress pH (Y) = 3.2, and salt stress concentration (Z) = 4 g/L KH_2_PO_4_. The protective effects of the stress treatments on MSJA during spray drying were ranked as follows: comprehensive treatment > heat shock > acid stress > salt stress. Acid stress and heat shock protect MSJA by reducing cell shrinkage, collapse, and volume loss, while salt stress aids in maintaining osmotic pressure balance across MSJA cell membranes during drying. This study not only optimized the stress treatment scheme of MSJA before spray drying, but also laid a foundation for both high efficiency and high quality spray drying MSJA. It also revealed the mechanism of each stress treatment, and provided guidance for the protection of other microorganisms by spray drying stress treatment.

**Supplementary Information:**

The online version contains supplementary material available at 10.1186/s40643-025-00939-9.

## Introduction

As an integral part of traditional Chinese culture, baijiu represents an invaluable intangible cultural heritage of the Chinese nation, with the baijiu industry acting as a pillar of the national economy (Cheng et al. 2022; Liu et al. 2023; Siddiqui et al. 2023; Ukić et al. 2021). Current research widely acknowledges that drying high-quality baijiu yeast to facilitate transportation, long-term storage, and large-scale promotion is crucial for improving baijiu brewing quality and advancing the liquor industry (Engeland et al. 2018, 2022; Dürr et al., 2020; Kang et al. 2025). Spray drying has emerged as a promising method for preserving high-quality liquor yeast, offering advantages such as high efficiency, automatic granulation, and low cost. However, the issue of low survival rates during drying remains a significant challenge (Al Zaitone et al. 2022; Cheng et al. 2021). MSJA, a baijiu yeast jointly developed by the China Baijiu Academy and Wuliangye Group, not only plays a pivotal role in enhancing the quality and flavour of baijiu brewing, but is also highly sensitive to strong acidity and high temperatures, making cost-effective transportation particularly difficult. As a representative example of high-quality baijiu yeast in China, optimising the stress treatment protection process for MSJA during spray drying and exploring the mechanisms behind each stress treatment using FESEM are essential for the cost-effective nationwide promotion of baijiu yeast and the rapid development of the baijiu industry.

Stress treatment is a process in which microorganisms are exposed to adverse conditions such as high temperatures, high osmolarity, strong acidity, and others, prior to drying (Matsumoto et al. 2022). This process triggers their innate defense mechanisms, leading to the production of protective components that improve their survival rate during drying. Currently, common stress treatment processes for edible microorganisms include heat shock, acid stress, salt stress, and starvation stress. Zhen et al. (2020) investigated the impact of heat shock treatment on the freeze-drying tolerance of *Lactobacillus acidophilus ATCC356* and its underlying mechanism, demonstrating that heat shock activated the bacterial defence mechanism, with the metabolic changes induced by the shock significantly improving its drying survival rate. Nadodkar et al.(2022) Took Oenococcus oeni and yeast as the research objects, explored its heat shock + freeze drying, and concluded that heat shock can enhance the stability of protein in yeast, further ensure the integrity of cells, so as to improve its drying survival rate and wine quality. Yang et al. (2023) examined the effect of heat shock treatment on the survival rate and fermentation ability of yeast during spray drying, revealing that heat shock not only improved yeast survival but also enhanced its fermentation capacity. Cnossen et al. (2020) explored the effects of acid and salt stress on the spray drying of *Lactococcus lactis*, showing that salt stress had minimal impact on survival rates. Gao et al. (2022) studied the survival rate of *Saccharomyces* yeast during freeze-drying under heat shock, acid stress, and starvation stress, finding that appropriate cross-stress treatments significantly improved survival rates. While the above studies have analyzed the effects of stress treatments on microbial drying survival, few have focused on response surface optimization. These studies tend to prioritise the glass transition temperature (storage resistance) as the optimisation goal, but fail to integrate culture conditions and stress treatments in relation to the large-scale production needs of enterprises and the growth of microbial cultures (Yuan et al. 2022).

In order to improve production efficiency and large-scale enterprise production, msja and other high-quality yeast are often cultivated, and then spray dried directly after adding protective agent and simple dehydration, without separate stress treatment process. Therefore, this study aims to balance high-efficiency, high-quality drying, normal-temperature, non-destructive storage, and convenient transportation and promotion, while ensuring large-scale, low-cost production. Initially, orthogonal and response surface methodology experiments were designed to optimise the processes of heat shock, acid stress, and salt stress, focusing on the growth of MSJA (OD600), survival rate (*Q*_*v*_) after drying, and glass transition temperature (*T*_*g*_). Subsequently, FESEM was employed to directly observe the changes in diameter, morphology, and cell wall microstructure of MSJA after spray drying under each stress treatment, revealing the protective effects and mechanisms of each process. Ultimately, this study aims to achieve high-efficiency, high-survival-rate, and large-scale continuous drying of MSJA, providing a valuable reference for stress treatments prior to microbial drying.

## Experimental design

### Experimental materials and experimental scheme design of spray drying

#### Experimental materials

In this study, the Pioneer LPG-5 spray dryer was chosen for the spray drying experiments. The main drying tower of the spray dryer had a height of h = 2 m, with a cylinder height of *h*_*1*_ = 1.14 m and a diameter of D = 800 mm. The effective drying section had a height of *h*_*2*_ = 1 m, a bottom conical taper of *a* = 60°, and an outlet aperture of *d* = 89 mm. The material was atomised using a motorised centrifugal nozzle of the DLP-50 type, featuring a spray disc diameter of 50 mm, a rated power of 0.55 kW, and a rated speed of 25,000 rpm (Xiong et al. 2024a, b).

The equipment utilised included an Ultraviolet-Visible Spectrophotometer (UV-Vis), Biological Optical Microscope (OM), Differential Scanning Calorimeter (DSC), Constant Temperature Shock Incubator, Moisture Content Tester, pH Meter, Autoclave, Vertical Flow Ultra-Clean Bench, Low-Temperature High-Speed Centrifuge, and Ultra-Low Temperature Freezer.

MSJA, a high-performance Baijiu yeast strain developed by the China Baijiu Institute in collaboration with Wuliangye Group from Sporidiobolus johnsonii, is inoculated onto fermentation substrates composed of wheat, barley, and pea to produce distiller’s yeast for Baijiu brewing. This strain significantly enhances the concentrations of key aromatic compounds such as n-propanol, 2-butanol, isoamyl alcohol, ethyl lactate, ethyl acetate, acetal, and furfural, thereby improving the richness, body, and aged character of the final Baijiu product. Distiller’s yeast incorporating MSJA has been widely adopted by major Baijiu producers partnered with the Baijiu Institute, contributing significantly to product quality and production efficiency. Given its industrial relevance, MSJA was selected as the model organism for investigating the spray drying of high-quality Baijiu yeast.

The NYEPD medium was developed by our team in a previous study, with the following formulation: sucrose molasses 51 g/L, beef paste 14 g/L, yeast paste 15 g/L, K_2_HPO_4_ 1.3 g/L, VB₁ 0.01 g/L, VB_2_ 0.01 g/L, and myo-inositol 0.01 g/L (Xiong et al. 2024a, b).

#### Spray drying experiment and detection process design

The MSJA spray drying experimental procedure consists of: cleaning → preheating → feeding → drying particles → 4 °C cold storage → testing and inspection. The parameters include a material flow rate of 700 mL/h, hot air flow rate of 70 m^3^/h, and an inlet hot air temperature of 120 °C.

The OD600 and *T*_*g*_ were determined using UV-Vis and DSC, while *Q*_*v*_ was measured using OM with the help of hematocrit (Xiong et al. 2024a, b).

### Optimised design of the stress treatment process before drying

#### Orthogonal experiments

(1) Orthogonal experimental design of heat shock.

In alignment with the optimisation requirements for the culture process parameters, the experimental optimisation scheme for the heat-excitation process parameters was designed as follows: ① The activated MSJA was inoculated in equal volumes into 15 NYEPD media, then incubated for 9 h at 180 rpm in a constant temperature and humidity incubator set to 28, 30, 32, 34, 36, and 38 °C, respectively. The pH of the incubation solution was measured at 2-hour intervals and maintained between pH 1 and 4 (Xiong et al. 2024a, b). ② Upon completion of the incubation, the OD600 of all samples was measured. ③ The culture medium, along with MSJA, was directly spray-dried under typical process conditions to obtain MSJA dry powder particles. Subsequently, *Q*_*v*_ and *T*_*g*_ were determined. ④ OD600, *Q*_*v*_, and *T*_*g*_ of the pellets were used as optimisation targets, and graphical analysis was conducted to determine the optimal temperature range for heat excitation.

(2) Orthogonal experimental design of acid stress.

For the optimisation of the acid stress process parameters, the experimental scheme was designed as follows: ① The activated MSJA was inoculated in equal volumes into 19 NYEPD media, then cultured for 9 h at a temperature of 34 °C and a rotational speed of 180 rpm in a constant-temperature, humidity, and shock incubator. The pH of the culture solution was measured at 0.5-hour intervals, with the control solution pH set at 1, 2, 3, 4, 5, and 6. ② After incubation, the OD600 of all samples was measured. ③ The culture medium, along with MSJA, was directly spray-dried under typical conditions to obtain MSJA dry powder particles, and *Q*_*v*_ and *T*_*g*_ were determined. ④ OD600, *Q*_*v*_ and *T*_*g*_ were used as optimisation targets, and graphical analysis was performed to determine the optimum pH range for acid stress. Salt stress differs from acid and thermal stress in that the type of inorganic salts added must be optimised experimentally before determining the optimal amount to add. Therefore, in this study, OD600 was used as an evaluation index to design experiments for the preliminary optimisation of the type and amount of inorganic salts. The experimental protocol was as follows: ① The inorganic salt component was removed from the NYEPD medium, and five types of inorganic salts—KH_2_PO_4_, CaCl_2_, MgSO_4_, ZnSO_4_, and NaCl—were added, each at concentrations of 2, 3, 4, 5, and 6 g/L, respectively. ② In a constant-temperature water-bath shaker incubator set at 34 °C, 70% humidity, and 180 rpm, the inorganic salts were added to the medium, and incubation was carried out for 9 h. ③ The OD600 of the samples was measured and analysed to identify the most suitable inorganic salt species.

(3) Orthogonal experimental design of Salt stress.

Salt stress is different from acid stress and thermal stress in that the type of added inorganic salts must also be experimentally optimized before experimental optimization of the amount of added inorganic salts; therefore, in this study, OD600 was used as an evaluation index to design experiments for the preliminary optimization of the type and amount of added inorganic salts. The experimental protocol was as follows:① After removing the inorganic salt component from the NYEPD medium, five kinds of inorganic salts, namely, KH_2_PO_4_, CaCl_2_, MgSO_4_, ZnSO_4_, and NaCl, were added, and the amount of each kind of inorganic salt was put into the medium was 2, 3, 4, 5 g/L, and 6 g/L, respectively.② In the constant-temperature water-bath shaker incubator at the temperature of 34 ℃, the humidity of 70%, and the rotation speed of 180 r/min, the inorganic salt was added into the medium, and the amount of each kind of inorganic salt was added into the medium. The incubation was carried out at 34 ℃, 70% humidity and 180 r/min speed for 9 h.③ The OD600 was measured and analyzed to find out the most suitable inorganic salt species.

The protocol for the optimisation experiment of homoeopathic salt stress parameters was as follows: ① The activated MSJA was inoculated in equal quantities into 15 NYEPD. ② The inorganic salts previously identified were added to the 15 media at concentrations of 3, 3.5, 4, 4.5, and 5 g/L, respectively. ③The cultures were incubated in a constant-temperature, humidity-controlled shaking incubator at 34 °C and 180 rpm for 9 h. The pH of the culture solution was measured at 2-hour intervals and was maintained between pH 2 and 4. ④ After the culture was completed, all samples were collected, and the OD600 was measured. ⑤ The culture medium, along with MSJA, was directly spray-dried under typical conditions to obtain MSJA dry powder particles, and *Q*_*v*_ and *T*_*g*_ were determined. ⑥ Graphical analysis was performed using OD600, *Q*_*v*_, and *T*_*g*_ as optimisation objectives to determine the optimal concentration range for the addition of inorganic salts.

#### Response surface experiments

Although the orthogonal experiments above provided an optimal range for the process parameters for heat excitation, salt stress, and acid stress, they did not yield the optimal values for all three processes simultaneously. Therefore, in this section, within the optimal ranges of the three process parameters, OD600, *Q*_*v*_, and *T*_*g*_ were once again used as the optimisation objectives. The temperature for heat stress (*X*), pH for acid stress (*Y*), and the concentration of added salt (*Z*) were treated as variables. A one-way experimental scheme was designed using the Box-Behnken Design model in Design Expert, with the results shown in Table 1.

**Table 1:** Results of one-way experiments

Serial number	X	Y	Z	OD600	Q_v_(%)	T_g_(°C)
1	36	4	4	2.66	53.5	47.2
2	35	3	4	2.7	53.9	47.4
3	35	3	4	2.7	53.9	47.4
4	34	3	3.5	2.67	53.6	47.2
5	35	2	4.5	2.68	53.7	47.3
6	36	3	4.5	2.68	53.7	47.3
7	35	3	4	2.7	53.9	47.4
8	34	4	4	2.65	53.4	47.1
9	35	3	4	2.7	53.9	47.4
10	35	2	3.5	2.68	53.7	47.3
11	36	2	4	2.67	53.6	47.2
12	36	3	3.5	2.68	53.7	47.3
13	35	4	3.5	2.67	53.6	47.2
14	34	3	4.5	2.67	53.6	47.2
15	34	2	4	2.65	53.4	47.1
16	35	4	4.5	2.67	53.6	47.2
17	35	3	4	2.69	53.8	47.3

Three replicate experiments were conducted based on the one-factor experimental protocol, and the average values were calculated and entered into the Box-Behnken Design module as required. After performing significance analysis, the results regarding the effects of *X*, *Y*, and *Z* on OD600, *Q*_*v*_, and *T*_*g*_ are presented in Table 2.

**Table 2:** Analysis of variance of regression model

Factor	Freedom	Mean square	F value	*P* value	Significance
model	9, 9	56.81, 59.15, 58.74	9.98, 11.64, 10.68	0.0003	**
*X*	1, 1	55.98, 60.65, 59.68	8.70, 9.85, 9.05	0.005, 0.004,0.014	**
*Y*	1, 1	47.78, 49.67, 48.98	14.29, 16.12, 16.21	0.029. 0.028, 0.015	**
*Z*	1, 1	99.00, 92.84, 91.26	14.65, 14.96, 14.86	0.850,0.740, 0.710	
*XY*	1, 1	12.22, 13.24, 12.87	6.65, 6.87, 7.20	0.007, 0.012, 0.009	**
*XZ*	1, 1	54.17, 55.36, 56.75	16.20, 15.98, 16.05	0.095, 0.046, 0.057	**
*YZ*	1, 1	43.40, 44.12, 48.61	14.01, 13.09, 14.07	0.085, 0.043, 0.087	
*X* ^2^	1, 1	34.92, 38.47, 35.9	10.34, 11.67, 12.97	0.004, 0.006, 0.016	**
*Y* ^2^	1, 1	50.57, 51.67, 52.15	12.12, 14.24, 13.98	0.008,0.009, 0.011	**
*Z* ^2^	1, 1	110.30, 112.65, 113.7	32.98, 35.36, 35.12	0.071, 0.094, 0.071	
Residual value	7, 7	3.34, 3.54, 3.49			
Anomalistic term	3, 3	7.14, 7.54, 7.62	14.36, 15.87, 16.01	0.673, 0.798, 0.067	
Pure error	4, 4	0.497, 0.524, 0.518			

Note: in the same table, “,” is used as the boundary. The former number represents OD600 as the target, ***Q***_***v***_ as the target and *T*_*g*_ as the target

**Fig. 1:**
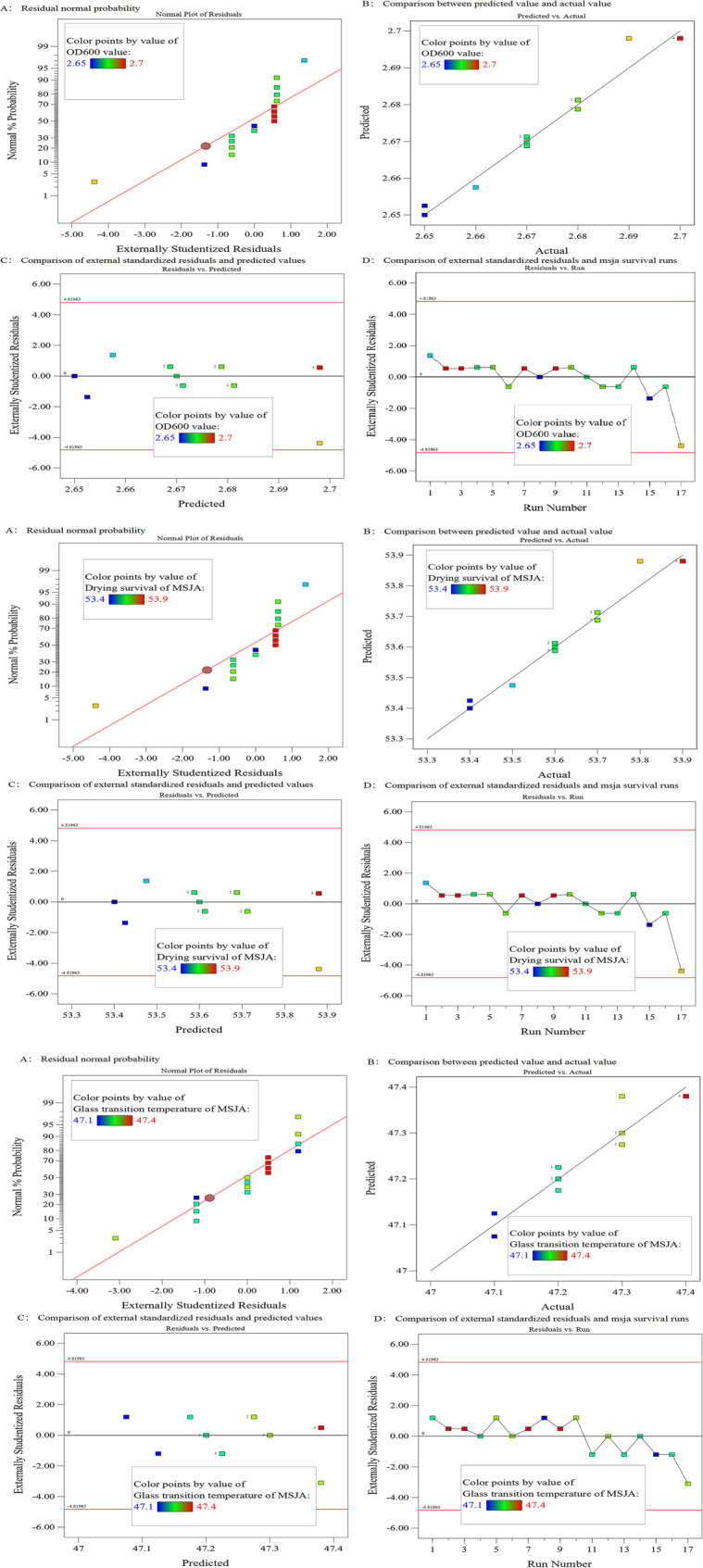
Diagnostic diagram of OD600, *Q*_*v*_, and *T*_*g*_ analysis

As presented in Table 2, when the objective is OD600, *P*_*X*_ = 0.005 < 0.01, *P*_*Y*_ = 0.029 < 0.05, and *P*_*Z*_ = 0.850 > 0.1. When the objective is *Qv*, *P*_*X*_ = 0.004 < 0.01, *P*_*Y*_ = 0.028 < 0.05, and *P*_*Z*_ = 0.740 > 0.1. When the objective is *T*_*g*_, *P*_*X*_ = 0.014 < 0.05, *P*_*Y*_ = 0.015 < 0.05, and *P*_*Z*_ = 0.710 > 0.1. Furthermore, *P*_*XY*_ are all less than 0.05, and only when the optimization target is *Q*_*V*_, *P*_*XZ*_ and *P*_*YZ*_ are less than 0.05, which is significant. In other cases, *P*_*XZ*_ and *P*_*YZ*_ are all greater than 0.05, which is not significant. These results indicate that the effects of changes in both *X* and *Y* were significant on OD600, *Q*_*v*_, and *T*_*g*_, while the effects of *Z* on OD600, *Q*_*v*_, and *T*_*g*_ were insignificant. Therefore, it is proposed that the preferred value of *Z* = 4 g/L, as calculated by Design Expert, be used as the default value, and a diagnostic evaluation be carried out for the two parameters, *X* and *Y*, targeting OD600, *Q*_*v*_, and *T*_*g*_. The results of this evaluation are shown in Fig. 1.

As presented in Fig. 1A, the experimentally determined values (coloured squares) are tightly clustered around the predicted values (diagonal line), indicating a normal distribution of both the model’s predicted values and the experimental data, which further confirms the independence and normality of the residuals. As presented in Fig. 1B, the determined values (coloured squares) align closely with the predicted values (line) and are uniformly distributed on both sides, demonstrating that the quadratic regression model provides an excellent fit for the response surface of the experimental data. Figure 1C shows that the predicted values (coloured squares) are centred around the 0-axis, with none exceeding the limit value (red line), confirming that the model’s predictive accuracy meets the required standards. Figure 1D indicates that the values from the model’s predictions are randomly and uniformly distributed near the 0-axis, without any discernible patterns or anomalies, further validating the model’s effectiveness and its suitability for optimizing the three stress parameters, *X*, *Y*, and *Z*. After confirming the model’s validity through diagnostic evaluation, response surface analysis for *X* and *Y* was optimised using the Box-Behnken Design, with OD600, *Q*_*v*_, and *T*_*g*_ as the objectives, as shown in Table 1.

### Experimental design of MSJA cell morphology observation

To elucidate the mechanisms by which different stress treatments increase the survival rate of MSJA during spray drying, the diameter, morphology, and cell wall structure of MSJA were directly observed using FESEM. The observation and testing procedure was as follows: ① The dried MSJA particles were attached to the sample stage using conductive tape. ② The samples were sprayed with gold at multiple angles using an ion sputter coater. ③The size distribution and cell wall morphology of MSJA at 5 µ m and 2 µ m resolution were observed by FESEM at 5 kV acceleration voltage. ④Observe and capture each area three times, then select the clearest and most representative image for analysis.

## Analysis and discussion of experimental results

### Analysis of orthogonal experiment

**Fig. 2:**
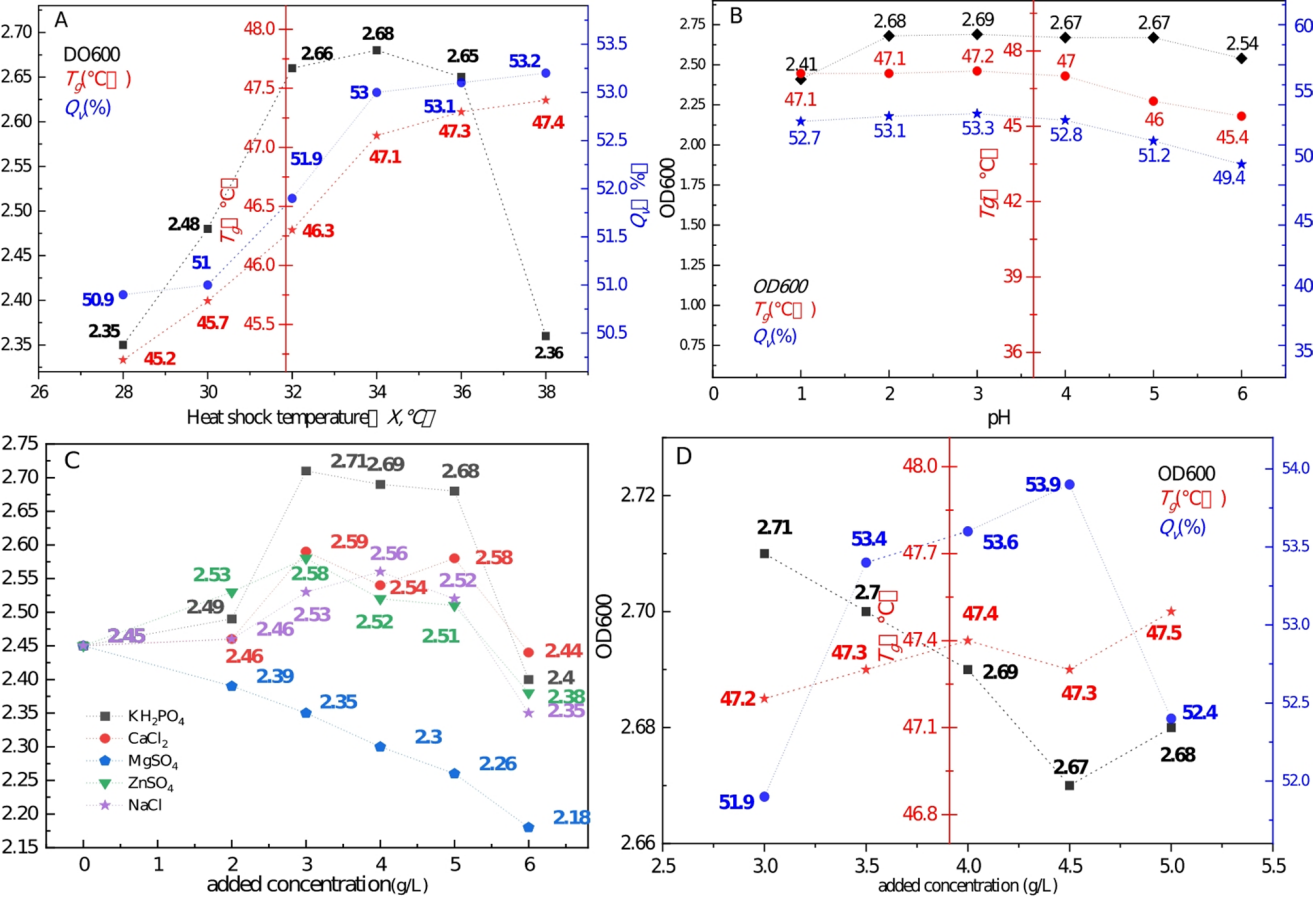
Effect of *X*, *Y*, and *Z* on OD600, *Q*_*v*_, and *T*_*g*_

The experiment was carried out according to the orthogonal experimental scheme. The obtained experimental data were plotted and analysed using Origin, and the resulting effects of *X*,* Y*, and *Z* on OD600, *Q*_*v*_, and *T*_*g*_ are shown in Fig. 2.

As illustrated in Fig. 2A, the OD600 value exhibited a sharp increase between 28 °C and 32 °C, then plateaued from 32 °C to 36 °C, reaching a peak of 2.68 at 34 °C. Beyond 36 °C, it decreased from 2.65 to 2.36. Therefore, based on OD600, the optimal incubation temperature range is 32 °C to 36 °C. The *Q*_*v*_ initially increased with temperature, peaking at 53% at 34 °C, after which it stabilised with a slow rise. Thus, for *Q*_*v*_ (drying protection), the optimal incubation temperature is also around 34 °C. When the temperature exceeded 36 °C, although high heat stress further stimulated the MSJA stress system to produce more heat shock proteins, this also hindered MSJA division, leading to a reduction in OD600. The trend for *T*_*g*_ mirrored that of *Q*_*v*_, indicating that the optimal temperature for storage, from a *T*_*g*_ perspective, lies between 34 °C and 36 °C. In summary, this experiment concluded that the optimal thermal excitation temperature range for MSJA is 34 °C to 36 °C.

Figure 2B shows the changes in OD_600_, where pH values between 1 and 2 led to an increase in OD600, followed by stability between 2.67 and 2.69 from pH 2 to 5. After pH > 5, OD600 began to decline. Therefore, the most suitable pH for MSJA culture is between 2 and 5. Regarding *T*_*g*_, before pH < 4, it remained stable between 47 °C and 47.2 °C. However, when pH > 4, *T*_*g*_ decreased steadily, suggesting that the optimal pH for MSJA storage is between 1 and 4. Analysis of *Q*_*v*_ revealed stability between 52.7% and 53.3% at pH 1–4, with a decline observed after pH > 4. Thus, the optimal pH for MSJA drying is also between 1 and 4. In conclusion, this experiment tentatively identified the optimal acid stress range for MSJA as pH 2 to 4.

As shown in Fig. 2C, the OD600 exhibited a pattern of increasing and then decreasing with the concentration of most inorganic salts, except for MgSO_4_, where the OD600 decreased directly as its concentration increased from 0. For the other salts, there was an inflexion point around 3 g/L or 4 g/L, where the OD600 transitioned from increasing to decreasing. When KH_2_PO_4_ was added at a concentration of 3 g/L, the OD600 reached its maximum value of 2.71, which was the highest among all the additives tested. The OD600 remained stable between 2.68 and 2.71 in the 3–5 g/L concentration range, but declined sharply when the concentration exceeded 5 g/L. Therefore, the experiment tentatively concluded that KH_2_PO_4_ is the preferred salt and the optimal concentration for MSJA growth is between 3 and 5 g/L.

As shown in Fig. 2D, the OD600 remained stable in the high range of 2.67–2.71 from the outset, as KH_2_PO_4_ concentration was optimised based on OD600 as the target during additive selection. The trend of *T*_*g*_ mirrored that of OD600, remaining steady in the 47.2–47.5 °C range. From the perspectives of both cultivation and storage, the applicable range of KH_2_PO_4_ addition concentration was concluded to be 3–5 g/L. Regarding *Q*_*v*_, it increased rapidly from 51.9 to 53.4% between KH_2_PO_4_ concentrations of 3 g/L to 3.5 g/L, and then stabilised between 53.4% and 53.9% within the 3.5 g/L to 4.5 g/L range. However, when the KH_2_PO_4_ concentration exceeded 4.5 g/L, *Q*_*v*_ sharply declined to 52.4% at 5 g/L. Therefore, from the perspective of *Q*_*v*_, the optimal concentration range for KH_2_PO_4_ addition is between 3.5 and 4.5 g/L. In conclusion, the experiment tentatively determined that the optimal KH_2_PO_4_ addition concentration range is 3.5–4.5 g/L.

### Analysis of response surface experiment

**Fig. 3:**
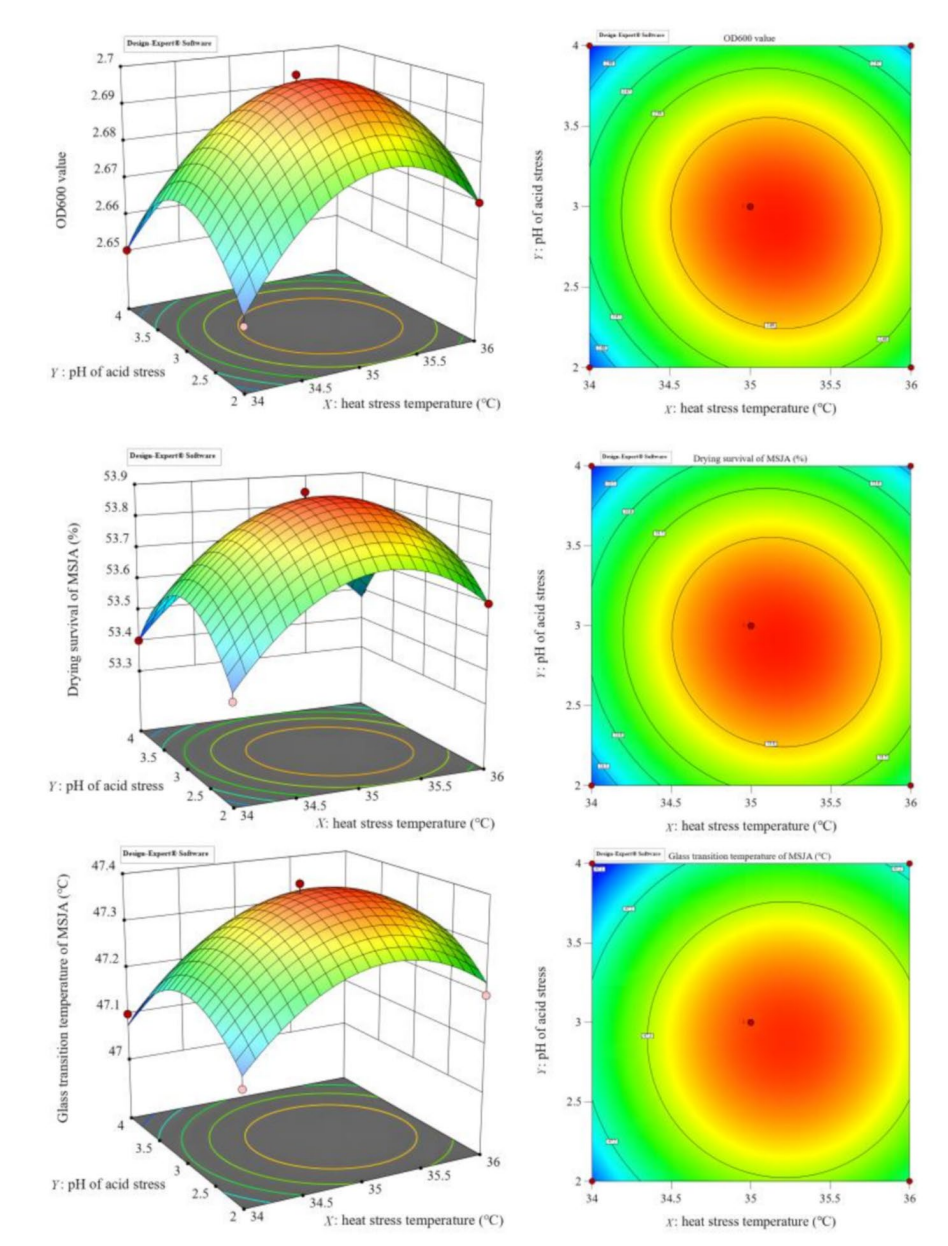
Response surface and contour map of *OD600*,* Q*_*v*_*and T*_*g*_

The experiments were conducted following the response surface experimental protocol, and the results are presented in Fig. 3.

As presented in Fig. 3, the positions marked with reddish-brown dots in the graphs represent the optimal balance of OD600, *Q*_*v*_, and *T*_*g*_, effectively supporting high-quality cultivation, enhanced viability during spray drying, and superior storage quality of MSJA. The optimisation equations for OD600, *Q*_*v*_, and *T*_*g*_, derived through response surface analysis, are presented in Eq. 1 below.

1 $$\begin{array}{l}\\\begin{gathered} OD600= - 0.02025{X^2} - 0.2025{Y^2} - 0.011{Z^2}\\- 0.0025X \cdot Y+2.085 \cdot {10^{ - 16}} \cdot X \cdot Z\\+2.167 \cdot {10^{ - 18}} \cdot Y*Z+1.4313X+0.2053Y+0.088Z+53.88 \\{Q_{ve}}= - 0.2025{X^2} - 0.2025{Y^2} - 0.011{Z^2} - \\0.0025X \cdot Y+2.073\cdot {10^{ - 15}} \cdot X \cdot Z +3.186 \cdot {10^{ - 17}} \cdot Y*Z+\\14.313X+2.052Y+0.88Z - 202.465 \\{T_C}= - 0.115{X^2} - 0.115{Y^2} - 0.06{Z^2} - 1.351 \cdot {10^{ - 14}}X \cdot Y+\\1.602 \cdot {10^{ - 15}} \cdot X \cdot Z \\ +\\1.047 \cdot {10^{ - 17}} \cdot Y*Z+8.1X+0.665Y+0.48Z - 97.165 \\ \end{gathered} \end{array}$$

Calculations based on Eq. 1 were performed to determine the optimal values for heat stress temperature (*X*), acid stress pH (*Y*), and KH_2_PO_4_ addition concentration (*Z*). The optimal values for cultivation (OD600) were *X* = 35.18 °C, *Y* = 3.18, and *Z* = 4. For dry survival rate (*Q*_*v*_), the optimal values were *X* = 35.19 °C, *Y* = 3.19, and *Z* = 4. For storage resistance (*T*_*g*_), the optimal values were *X* = 35.18 °C, *Y* = 3.19, and *Z* = 4.

After optimisation with OD600, *Q*_*v*_, and *T*_*g*_ as the objectives, the optimal values for thermal stress temperature (*X*), acid stress pH (*Y*), and KH_2_PO_4_ addition concentration (*Z*) were determined to be 35.2 °C, 3.2, and 4 g/L, respectively, rounded to one decimal place (since the thermometer and pH meter display values to one decimal place). Therefore, based on the response surface analysis, the optimal stress treatment process parameters for MSJA spray drying were: thermal stress temperature (*X*) = 35.2 °C, acid stress pH (*Y*) = 3.2, and KH_2_PO_4_ concentration for salt stress (*Z*) = 4 g/L.

MSJA was subjected to stress under these optimal process parameters, and the survival rate was assessed through spray drying tests. The results were as follows: comprehensive stress treatment (heat stress + acid stress + salt stress) 54.8% >heat stress 49.7% >acid stress 48.5% >salt stress 43.6% >no treatment 38.5%.

### FESEM observation and experimental analysis

The latest research by Resende et al. (2025) found that the survival rate of freeze-dried Pichia kluyveri CCMA 0615 and *Saccharomyces* cerevisiae CCMA 0732 was higher following heat shock and acid stress treatments, with the resulting cells being smaller and more uniformly distributed. This suggests that heat shock and acid stress may enhance drying survival rates by influencing yeast morphology and size. Based on these findings, this study also investigated the mechanism of stress treatment, focusing on the effects of heat shock, acid stress, and salt stress on the size, morphology, and cell wall structure of MSJA after drying.

**Fig. 4:**
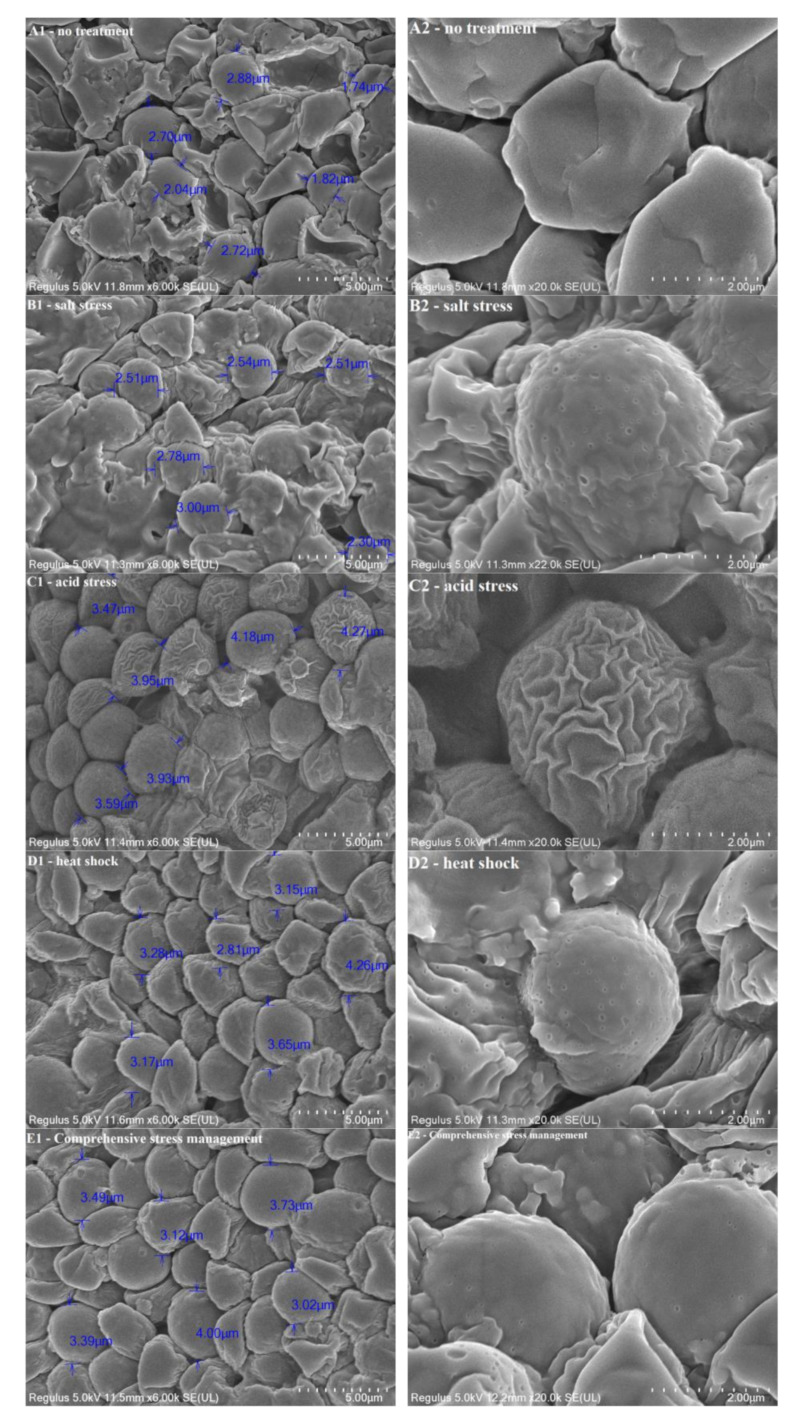
Diameter, morphology, and cell wall microstructure of MSJA after drying under various stress treatments

FESEM was used to observe the diameter, morphology, and cell wall microstructure of MSJA cells after spray drying under various stress treatments, including no treatment, heat shock, acid stress, salt stress, and comprehensive stress, as shown in Fig. 4.

From the comparison of A to E in Fig. 4, the following observations can be made: ① The average diameter of MSJA cells after drying was ranked as follows: acid stress > comprehensive treatment > heat shock > salt stress > no treatment. ② The uniformity of the diameter distribution followed this order: comprehensive treatment > heat shock > acid stress > salt stress > no treatment. ③ The severity of cell wall shrinkage and collapse increased in the following order: comprehensive treatment < heat shock < acid stress < salt stress < no treatment.

### Discussion

By comparing the survival rate of MSJA after drying with the FESEM observations, the protective mechanisms of each stress treatment process on the MSJA drying process can be summarised as follows:

① Compared to no treatment, the average diameter of MSJA after drying under heat shock and acid stress increased significantly, while the shrinkage and collapse of the cells were markedly reduced. Simultaneously, the survival rate after drying rose sharply from 38.5 to 49.7% and 48.5%, respectively. This suggests that heat shock and acid stress may mitigate the shrinkage, collapse, and volume reduction of MSJA cells, ultimately facilitating high-quality drying protection. Yilmaz et al. (2024) investigated the effects of heat shock, salt stress, and oxygen stress on *Saccharomyces* cerevisiae NCYC 88 and NCYC 79, concluding that heat shock reduces cell wall and membrane damage by promoting the synthesis of heat shock proteins and trehalose, thereby achieving drying protection. Liu et al. (2015) explored the impact of acid stress on the growth and fermentation efficiency of Freddo wine yeast, finding that acid stress at pH 2.5–4.5 promotes the synthesis of unsaturated fatty acids and antioxidant enzymes, enhancing both drying protection and fermentation. Therefore, acid stress and heat shock regulate the fluidity and stability of the MSJA cell wall and membrane by stimulating the synthesis of unsaturated fatty acids, antioxidant enzymes, heat shock proteins, and trehalose, ultimately ensuring effective drying protection for MSJA.

② Compared to no treatment, the average diameter of MSJA after drying under salt stress showed only a slight increase, and the shrinkage and collapse of cells remained evident. However, the survival rate after drying increased from 38.5 to 43.6%. This suggests that salt stress treatment did not effectively protect MSJA from drying damage by mitigating cell shrinkage, collapse, or volume reduction. Nadodkar et al. (2024) conducted salt stress experiments on Gubpc1 yeast in India and concluded that salt stress protected the yeast by maintaining osmotic pressure balance across the cell membrane. Similarly, Chen et al. (2024) confirmed the same mechanism in their review of salt stress treatments for microbial drying protection. In conclusion, salt stress works by allowing small molecular salts to freely enter and exit the cell membrane, thereby regulating osmotic pressure inside and outside the MSJA cells, thus completing the drying protection process for MSJA.

## Conclusion

This study comprehensively addressed the needs of large-scale industrial production by integrating stress treatment with cultivation processes. An orthogonal experiment and response surface experiment were designed to optimize the process. Focusing on the survival rate and storage quality of MSJA after cultivation and drying, with OD600, *Q*_*v*_, and *T*_*g*_ as the objectives, the heat shock temperature, acid stress pH, and salt concentration were optimized. The results showed that the optimal process parameters for stress treatment of MSJA yeast solution in the process of culture were: heat shock temperature *X* = 5.2 ℃, acid stress pH *Y* = 0.2, and salt stress concentration Z = 4 g/L with KH_2_PO_4_. At the same time, further analysis showed that the protection mechanism of each stress treatment process was that acid stress and heat shock could regulate the fluidity and stability of MSJA cell wall and cell membrane by promoting the synthesis of unsaturated fatty acids, antioxidant enzymes, heat shock protein and trehalose, and finally slow down the shrinkage and collapse of MSJA cells to complete the drying protection of MSJA. Salt stress can protect MSJA by regulating osmotic pressure inside and outside MSJA cells through free entry and exit of small molecular salt.

This study not only optimized the optimal parameters of heat shock, acid stress and salt stress before msja drying, increased the survival rate of msja spray drying from 38.5 to 54.8%, and extended the shelf life at room temperature by 12%, which promoted the large-scale promotion of high-quality baijiu yeast such as MSJA, and promoted the high-quality development of the Baijiu industry. It also revealed the protective mechanism of heat shock, acid stress and salt stress on msja drying, which provided guidance for the research on drying stress treatment of other yeast and other microorganisms.

## Supplementary Information

Below is the link to the electronic supplementary material.

Supplementary Material 1

## Data Availability

All data generated or analyzed during this study are included in this published article.

## Abbreviations

MSJA: Modified Sporidiobolus Johnsonii A
FESEM: Field Emission Scanning Electron Microscope
OD600: The growth of MSJA
*Q*_*v*_: Survival rate after drying
*T*_*g*_: Glass transition temperature after drying
UV-Vis: Ultraviolet-Visible Spectrophotometer;
OM: Biological Optical Microscope;
DSC: Differential Scanning Calorimeter;
*X*: Temperature of heat stress
*Y*: pH of acid stress
*Z*: Concentration of salt added
NYEPD: Our team in the previous study developed Medium
