# Supplementary figures and images for: Optimization of heat shock, acid shock and salt stress process and its mechanism of protection before spray drying of Baijiu yeast - Modified sporidiobolus Johnsonii A
Source: Bioresour Bioprocess. 2025 Nov 5;12(1):129. doi: 10.1186/s40643-025-00939-9 (PMC12589692; doi:10.1186/s40643-025-00939-9)

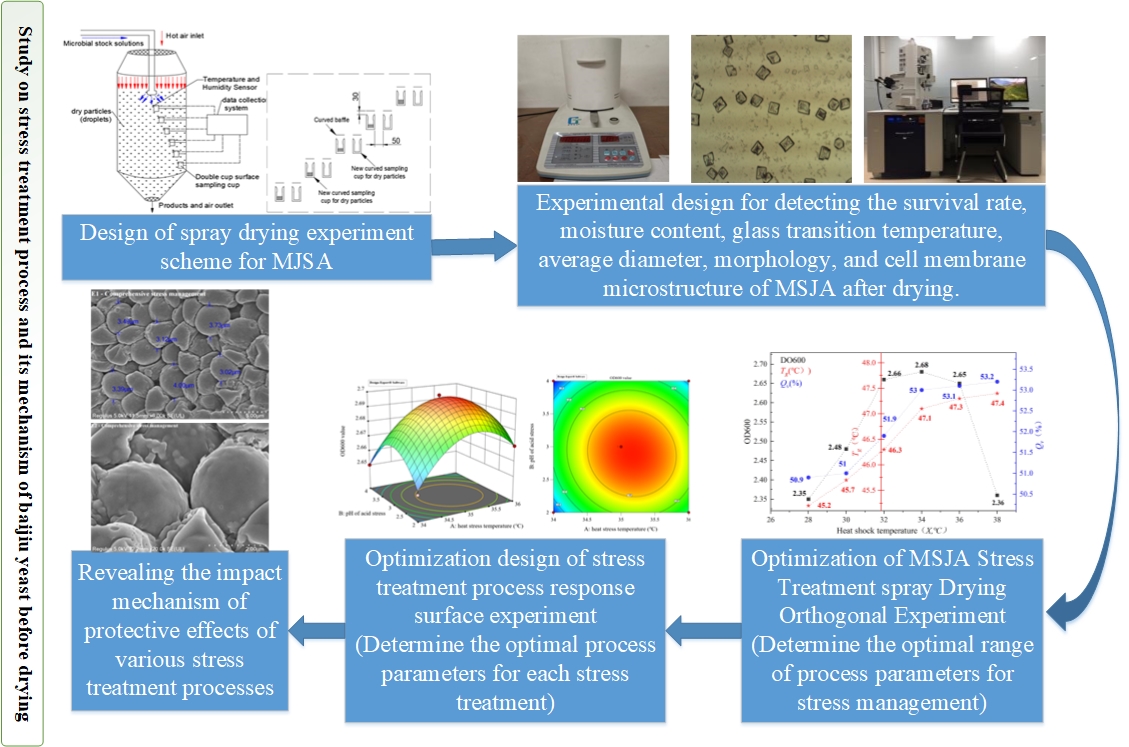

Supplement: Supplementary file 1 — Supplementary Material 1 [file 40643_2025_939_MOESM1_ESM.jpg]
